# In-Stent Restenosis in Peripheral Arterial Disease: Ultra-High-Resolution Photon-Counting Versus Third-Generation Dual-Source Energy-Integrating Detector CT Phantom Study in Seven Different Stent Types

**DOI:** 10.1007/s00270-024-03874-y

**Published:** 2024-11-05

**Authors:** Theresa-Marie Dachs, Sven R. Hauck, Maximilian Kern, Catharina Klausenitz, Maximilian Hoffner, Melanie Schernthaner, Hanaa Abdel-Rahman, Albert Hannover, Andreas Strassl, Irene Steiner, Christian Loewe, Martin A. Funovics

**Affiliations:** 1 Department of Bio-Medical Imaging and Image-Guided Therapy, Division of Cardiovascular and Interventional Radiology, Medical University of Vienna, Waehringer Guertel 18-20, 1090 Vienna, Austria; 2 Center for Medical Data Science, Institute of Medical Statistics, Medical University of Vienna, Vienna, Austria

**Keywords:** In-stent restenosis, Ultra-high-resolution photon-counting detector CT, Energy-integrating detector CT, Phantom study

## Abstract

**Purpose:**

The visualization of peripheral in-stent restenosis using energy-integrating detector CT is challenging due to deficient spatial resolution and artifact formation. This study compares the first clinically available photon-counting detector CT to third-generation dual-source energy-integrating detector CT.

**Materials and Methods:**

Nylon cylinders with central bores (4 mm, 2 mm), mimicking 75% and 95% stenoses, were placed inside seven different 8-mm diameter stents and filled with diluted contrast medium. Phantoms were scanned with photon-counting detector CT at slice thicknesses of 0.2 mm (available only in this scanner type), 0.5 mm, and 1.0 mm versus 0.5 mm and 1.0 mm in energy-integrating detector CT at matched CT dose indices. Contrast-to-noise ratios were calculated from attenuation rates. Residual lumen size was measured as full width at half-maximum. Subjective image quality was assessed by two independent blinded raters.

**Results:**

Mean contrast-to-noise ratio was lowest in photon-counting detector CT at 0.2 mm slice thickness (0%, 75%, and 95% in-stent restenosis: 6.11 ± 0.6, 5.27 ± 0.54, and 5.02 ± 0.66) and highest at 1.0 mm slice thicknesses with similar measurements in photon-counting detector CT and energy-integrating detector CT (11.46 ± 1.08, 9.94 ± 1.01, 8.26 ± 1.0 vs. 3.34 ± 1.0, 9.92 ± 0.38, 7.94 ± 1.07). Mean full width at half-maximum measurements in photon-counting detector CT at 0.2 mm slice thickness for 0%, 75%, and 95% in-stent restenosis were 8.00 ± 0.37, 3.98 ± 0.34, and 1.92 ± 0.16 mm. Full width at half-maximum was least precise in 95% in-stent restenosis at 1.0 mm slice thickness with similar measurements between scanners (1.57 ± 0.33 vs. 1.71 ± 0.15 mm). Interrater correlation coefficient was 0.75 [95% CI: [0.53; 0.86]; subjective scores were best at 0.2 mm slice thickness in photon-counting detector CT (19.43 ± 0.51 and 19.00 ± 0.68).

**Conclusion:**

In phantom in-stent restenosis in 8 mm stents, we observed similar full width at half-maximum for photon-counting detector CT and energy-integrating detector CT in 0% and 75% in-stent restenosis, but at 95% in-stent restenosis, FWHM tended to be more accurate in smaller slice thicknesses in both scanners. Subjective image assessment yielded best results at 0.2 mm slice thickness in photon-counting detector CT despite lower contrast-to-noise ratio.

**Graphical Abstract:**

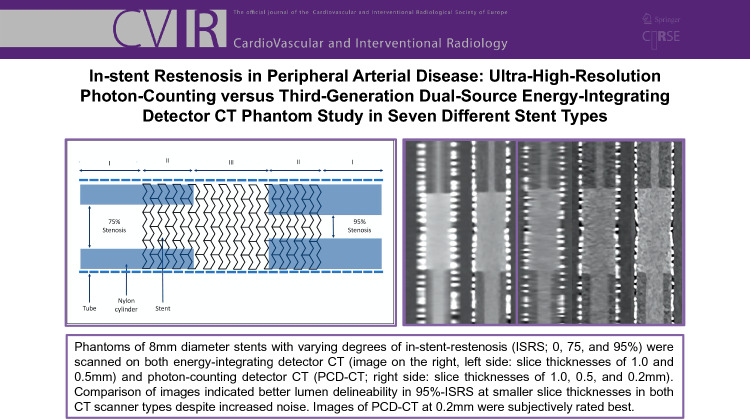

**Supplementary Information:**

The online version contains supplementary material available at 10.1007/s00270-024-03874-y.

## Introduction

Until the introduction of photon-counting detectors (PCD) in late 2021 [1], energy-integrating detectors (EID) were the only available CT detectors. These EID use a scintillator to convert incoming X-ray photons to visible light. In a second step, the light is converted to an electrical signal by a photodiode. The detector elements are separated by reflective septa to channel the incoming light and minimize cross-talk between neighboring detector elements and the sum of X-ray energy reaching each detector element during the measuring interval results in the final signal.

In contrast, PCD use a semiconductor layer that generates an electronic signal from incoming X-ray photons directly (without the need for conversion to visible light and accordingly, reflective septa). In PCD, X-ray photons are furthermore weighed by their inherent energies and generate individual proportional electrical signals [2, 3].

These differences explain important advantages of PCD- over EID-CT technology: With no need for separating septa, PCD enable smaller pixel sizes and higher spatial resolution images such as improved patient radiation dose efficiency. By weighing individual X-ray photonic energies, low-energy electronic noise can be subtracted, which leads to improved contrast-to-noise ratio (CNR), as well as interfering signals such as beam hardening and blooming artifacts. Besides, spectral imaging similar to dual-energy CT is possible (by definition and use of energy thresholds–so-called ‘bins’ [4–7].

Utilizing these advantages, PCD-CT can improve detection accuracy in peripheral stents in CT angiography (CTA) regarding limiting beam-hardening artifacts [8, 9]. When an in-stent restenosis (ISRS) is assessed, hemodynamically relevant lesions and low-grade stenoses should be differentiated first; second, a clear differentiation between high-grade stenoses and occlusions is desirable due to the associated therapeutic consequences [10, 11]. Traditionally, conventional CT technology has been associated with limitations regarding ISRS detection, such as artifacts and spatial resolution [12, 13].

Recent phantom studies tested stent visualization and in-stent lumen delineability on the first clinically applicable PCD-CT and found overall superior image quality of stented phantoms using ultra-high-resolution (UHR) PCD-CT [14–16]. However, data about more complex phantoms simulating ISRS (as have been demonstrated in investigational PCD-CT [7, 17]) are missing. Currently, the reliability of the most recent PCD-CT technology regarding the differentiation between residual lumen and ISRS has not been tested.

The purpose of the present study was 1) to evaluate and compare clinically applied CTA protocols using UHR PCD-CT versus third-generation dual-source EID-CT regarding the detectability of peripheral artery ISRS and 2) to test this protocol on high- and low-grade stenoses in a phantom using seven commonly used peripheral arterial stent types.

## Materials & Methods

### Phantom and Stents

**Fig. 1:**
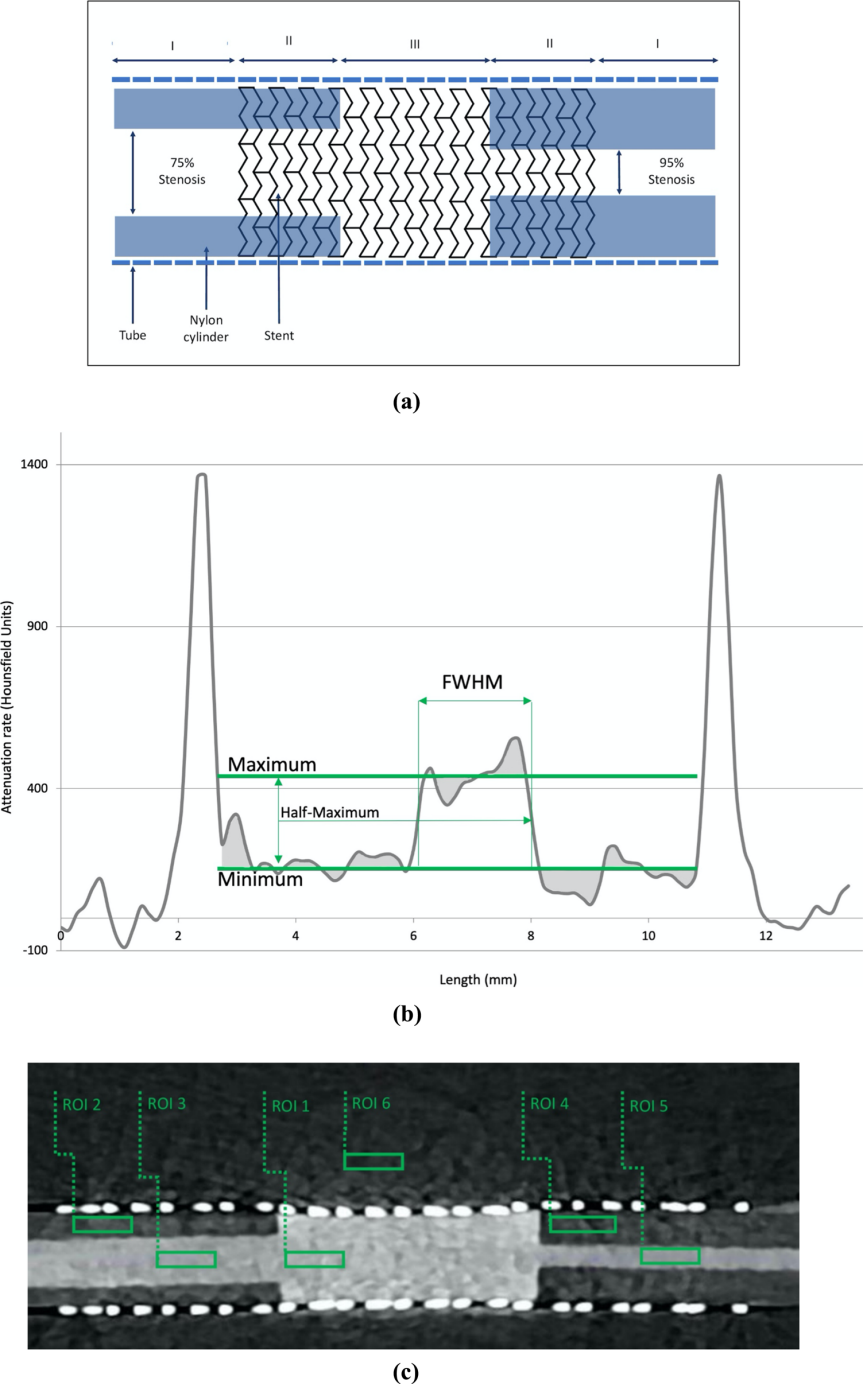
**a** Peripheral artery in-stent restenosis phantom. I = Stenosis outside stent; II = stenosis inside stent; III = stent without stenosis. Stents with a diameter of 8 mm (length varying between 40 and 60 mm) were placed inside plastic tubes (outer diameter = 10 mm, inner diameter = 8.5 mm). Cylindrical solid silicone stenosis (length = 30 mm, diameter = 8 mm) with residual lumina of 4 and 2 mm, respectively, was placed on opposite sides of the stent. Tubes were filled with diluted contrast medium, mimicking contrast-enhanced blood and sealed with silicone. Phantoms were then placed inside a water-filled container measuring 36 × 26 × 14 cm, imitating surrounding tissue. **b** Measurement of full width at half-maximum in a plot profile of a 95% in-stent restenosis. FWHM = full width at half-maximum. The maximum and minimum (average by area) of the plot profile were measured to identify the half-maximum. FWHM is measured between the crossings of the curve at half-maximum. **c** Image of in-stent restenosis phantom using ultra-high-resolution photon-counting detector computed tomography. ROI = region of interest. Attenuation rates of six specific ROI were measured: inside the non-stenosed stented lumen (ROI 1); inside the 75% stenosis of the stented segment (ROI 2); inside the lumen of the 75% stenosed stented segment (ROI 3); inside the 95% stenosis of the stented segment (ROI 4); inside the lumen of the 95%-stenosed stented segment (ROI 5); and the standard deviation of density outside the phantom indicating image noise (ROI 6)

Seven different stent models with a uniform lumen size of 8 mm and variable length (between 40 and 60 mm, see Supplemental Table I) typically used in the clinical routine were positioned inside polyethylene tubes with a length of 110 mm, an inner diameter of 8.5 mm and an outer diameter of 10 mm. Solid nylon cylinders (with a density that approximates the density of neointima) with a diameter of 8 mm were cut into pieces of 30 mm and bored centrally through the longitudinal axis. For each model, two solid cylindrical nylon stenoses with four- and two-mm bores, representing approximately 75% and 95% stenoses, respectively, were manufactured. The artificial stenoses were placed inside opposite ends of the stent, partly inside the stented area of the phantom, and partly outside, to enable measurements of stenosed and non-stenosed segments inside the stent. The phantoms were sealed with silicone on both sides and filled with a mixture of contrast media and saline (mimicking contrast-enhanced blood) using hypodermic needles. All phantoms were manufactured by the authors. The phantoms were placed inside a saline-filled container measuring 36 × 26 × 14 cm (length x width x height), as previously demonstrated [15]. The phantoms were placed in a central position, normal to the longitudinal axis of the gantry. A diagram of the phantom setup is depicted in Fig. 1a.

The stent models used are described in Supplemental Table I. The contrast agent used was Iomeron 400 (400 mg J/ml, Bracco Imaging, Milan, Italy). To mimic the physiologic absorption rate of contrast-enhanced blood (i.e., approximately 400 HU at 120 kV), a dilution series was performed to determine the correct ratio of 2.5% contrast media in saline. Before imaging, the phantoms were centrifuged to collect the remaining air at the top and de-aired in a vacuum chamber.

### CT Angiography

Images were obtained with a first-generation PCD-CT (NAEOTOM Alpha, Siemens Healthineers, Forchheim, Germany) with a dose-optimized protocol, in UHR. The following scan parameters were used: collimation 120 × 0.2 mm; tube voltage 120kVp; tube current time product 200 mAs; rotation time 500 ms; and pitch factor 0.55. For image reconstruction, three degrees of slice thickness (0.2 mm, 0.5 mm, 1.0 mm), a Bv76 vascular kernel, a matrix size of 1024 × 1024, and an image field-of-view adapted to the size of the phantom were used, resulting in an in-plane pixel size of 0.14 × 0.14 mm.

Reference images were obtained with a third-generation dual-source EID-CT (SOMATOM Force, Siemens Healthineers, Forchheim, Germany). The acquisition parameters applied were collimation 192 × 0.6 mm; tube voltage 120kVp; tube current time product 240mAs; rotation time 500 ms; and pitch factor 0.55. Parameters were selected to match the dose applied in the PCD-CT acquisition using CT dose index (CTDI_vol_; 16 mGy) [18]. For image reconstruction, slice thickness was set to a minimum of 0.5 mm and to 1.0 mm, a Bv59 vascular kernel, a matrix size 512 × 512, and image field-of-view was adapted to the size of the phantom, resulting in a pixel size of 0.35 × 0.35 mm.

### Quantitative Image Analysis

Sixteen-bit DICOM images were analyzed using the open-access software ImageJ [19]. For calculation of full width at half-maximum (FWHM), rectangular ROIs were created and positioned perpendicular to the longitudinal stent axis over the non-stenosed stented segment and the 75% and the 95% stenosis, respectively. Along the length of the ROI, mean pixel intensities were measured and plotted over the cross section. Full width at half-maximum (FWHM) was calculated from the resulting data plots (Fig. 1b).

Furthermore, the attenuation rates (in Hounsfield Units, HU) of six specific regions of interest (ROI) were measured (Fig. 1c): inside the non-stenosed stented lumen (ROI 1); inside the 75% stenosis of the stented segment (ROI 2); inside the lumen of the 75%-stenosed stented segment (ROI 3); inside the 95% stenosis of the stented segment (ROI 4); inside the lumen of the 95%-stenosed stented segment (ROI 5); and the standard deviation of density outside the phantom indicating image noise (ROI 6). ROIs were measured with fixed sizes and at the same location for each individual stent phantom. Mean values of each ROI were calculated from three consecutive measurements. Derived parameters were calculated as previously demonstrated [7]: (a) the CNR of the non-stenosed stented segment [(ROI1–ROI4)/ROI6], (b) the 75% stenosed stented segment [(ROI3–ROI2)/ROI6], and (c) the 95% stenosed stented segment [(ROI5–ROI4)/ROI6]. Image analysis, measurements and described calculations were performed by the authors.

### Qualitative Analysis

The delineability of ISRS and residual lumen in axial and longitudinal multiplanar reformations was evaluated by two authors (T.D, M.F. 1 and 26 years of experience, respectively) regarding 4 distinct qualities: sharpness, subjective image noise, blooming, and diagnostic confidence. To each of these qualities, a score of 1–5 was assigned (1 = non-diagnostic, 5 = excellent) as described previously [7, 14]. A sum score was calculated for each combination of scanner type and slice thickness.

### Statistical Evaluation

Statistical analyses were carried out by the authors using R 4.2.2 [20]. Quantitative variables are summarized as mean ± standard deviation.

Data were graphically illustrated in the form of boxplots. Comparison of stent models at different slice thicknesses (0.2 mm, 0.5 mm, 1.0 mm), stenosis grade (0%, 75%, 100%) and CT type (PCD-CT, EID-CT) was done by descriptive statistics. Bland–Altman plots were plotted to analyze the agreement between two consecutive measurements of FWHM. An interrater correlation coefficient (ICC) with 95% confidence limits (R-package irr, R-function icc) [21] was calculated based on a two-way random effects model to test for the agreement of qualitative measurements performed by two raters who assessed images independently and were blinded to scanner type such as slice thickness. ICC values less than 0.5 indicate poor, values between 0.5 and 0.75 moderate, values between 0.75 and 0.9 good, and values greater than 0.9 excellent reliability [22].

## Results

### Contrast-to-Noise Ratio (CNR)

Stratified by stent model, observed mean CNR was highest and therefore best in self-expandable nitinol stents, followed by the balloon-expandable stainless steel and cobalt-chromium alloy stents. Respective measurements are displayed in Supplemental Table II.

**Fig. 2:**
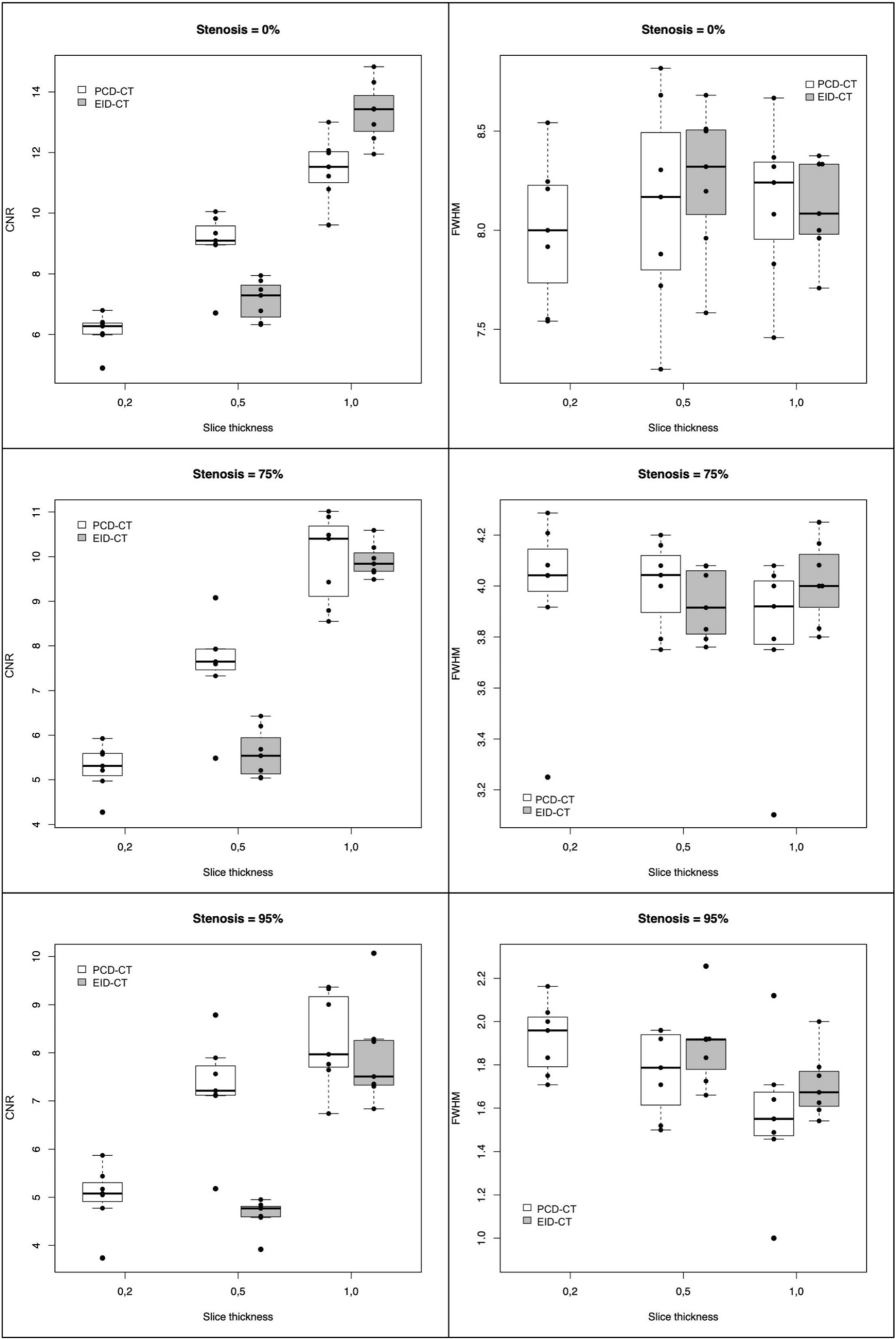
Boxplots of contrast-to-noise ratio and full width at half-maximum measurements at 0%, 75% and 95% stenosis EID-CT = energy-integrating detector computed tomography; FWHM = full width at half-maximum; PCD-CT = photon-counting detector computed tomography; CNR = contrast-to-noise ratio. White boxplots represent PCD-CT measurements, whereas gray boxplots represent measurements of EID-CT. The box shows the first quartile (bottom), median (bold line within the box) and the third quartile (top). Whiskers extend to the minimum and maximum. Outliers are defined as observations that are outside the interval [first quartile–1.5 × interquartile range; third quartile + 1.5 × interquartile range]. Outliers are shown as circles and in the case of outliers, the whiskers extend to the smallest/largest observation within that interval. In addition, the individual data points are shown in the graphics. Coincident points are stacked

Across all stent types, observed mean CNR was highest in the non-stenosed stented segment, followed by the 75%-stenosed segment, and lowest in the 95%-stenosed segment. CNR increased with greater slice thickness in both PCD-CT and EID-CT. Compared to EID-CT, PCD-CT displayed an overall higher CNR at a slice thickness of 0.5 mm and a similar CNR in the 75%- and 95%-stenosed segments at 1.0 mm slice thickness. Respective results are depicted in Table 1 and depicted as boxplots in Fig. 2.

**Table 1:** Contrast-to-noise ratio measurements of in-stent restenosis in PCD-CT and EID-CT at varying slice thicknesses

Slice thickness, mm	Stenosis, %	PCD-CT		EID-CT	
		Mean	SD	Mean	SD
0.2	**0**	6.11	0.60	–	–
	**75**	5.27	0.54	–	–
	**95**	5.02	0.66	–	–
0.5	**0**	8.99	1.09	7.14	0.66
	**75**	7.57	1.08	5.59	0.55
	**95**	7.27	1.10	4.64	0.34
1.0	**0**	11.46	1.08	13.34	1.00
	**75**	9.94	1.01	9.92	0.38
	**95**	8.26	1.00	7.94	1.07

Number of observations = 7 in each setting

EID-CT = energy-integrating detector computed tomography; PCD-CT = photon-counting detector computed tomography; SD = standard deviation

### Full width at half-maximum (FWHM)

Agreement between two consecutive measurements of FWHM analyzed by Bland–Altman plots is shown in Supplemental Figure I. Regarding mean FWHM measurements of non-stenosed stented segments, referred to as 0% stenosis, the 95% confidence interval of the mean (CI) included the true stent diameter of 8 mm in all combinations of scanner type and slice thickness, at 0.2 mm, 0.5 mm, and 1.0 mm in PCD-CT with respective measurements of 8.00 ± 0.37 mm, 8.12 ± 0.54 mm, and 8.14 ± 0.40 mm, such as in EID-CT at slice thicknesses of 0.5 mm and 1.0 mm with 8.25 ± 0.38 mm and 8.11 ± 0.25 mm.

FWHM measurements of 75%-stenosed stented segments were similarly close to the true lumen of 4 mm in both PCD-CT and EID-CT at different slice thicknesses. In PCD-CT, at respective slice thicknesses of 0.2 mm, 0.5 mm, and 1.0 mm, mean FWHM was measured at 3.98 ± 0.34 mm, 4.00 ± 0.17 mm, and 3.81 ± 0.34 mm. In comparison, EID-CT at slice thicknesses of 0.5 and 1.0 mm yielded mean FWHM measurements of 3.93 ± 0.14 mm and 4.02 ± 0.16 mm.

Mean FWHM measurements at 95%-stenosed stented segments for PCD-CT (0.2, 0.5, and 1.0 mm slice thickness) were 1.92 ± 0.16 mm, 1.76 ± 0.2 mm, and 1.57 ± 0.33 mm, compared to EID-CT (0.5 and 1.0 mm slice thickness) with 1.89 ± 0.19 mm and 1.71 ± 0.15 mm. In PCD-CT at a slice thickness of 0.5 mm and 1.0 mm and EID-CT at a slice thickness of 1.0 mm, the upper limit of the CI was below the true lumen diameter in 95%-stenosed stented segments. Respective data are shown in Table 2 and Fig. 2.

**Table 2:** Full width at half-maximum measurements of in-stent restenosis in PCD-CT and EID-CT at varying slice thicknesses

Slice thickness, mm	Stenosis, %	PCD-CT			EID-CT		
		Mean	SD	CI	Mean	SD	CI
0.2	**0**	8.00	0.37	7.66; 8.34	–	–	–
	**75**	3.98	0.34	3.66; 4,29	–	–	–
	**95**	1.92	0.16	1.77; 2.07	–	–	–
0.5	**0**	8.12	0.54	7.63; 8.62	8.25	0.38	7.90; 8.6
	**75**	4.00	0.17	3.84; 4.16	3.93	0.14	3.80; 4.06
	**95**	1.76	0.20	1.58; 1.95	1.89	0.19	1.71; 2.07
1.0	**0**	8.14	0.40	7.77; 8.5	8.11	0.25	7.88; 8.34
	**75**	3.81	0.34	3.5; 4.12	4.02	0.16	3.87; 4.17
	**95**	1.57	0.33	1.26; 1.88	1.71	0.15	1.57; 1.85

Number of observations = 7 in each setting

CI = 95% confidence interval; EID-CT = energy-integrating detector computed tomography; PCD-CT = photon-counting detector computed tomography; SD = standard deviation

### Qualitative Analysis

**Fig. 3:**
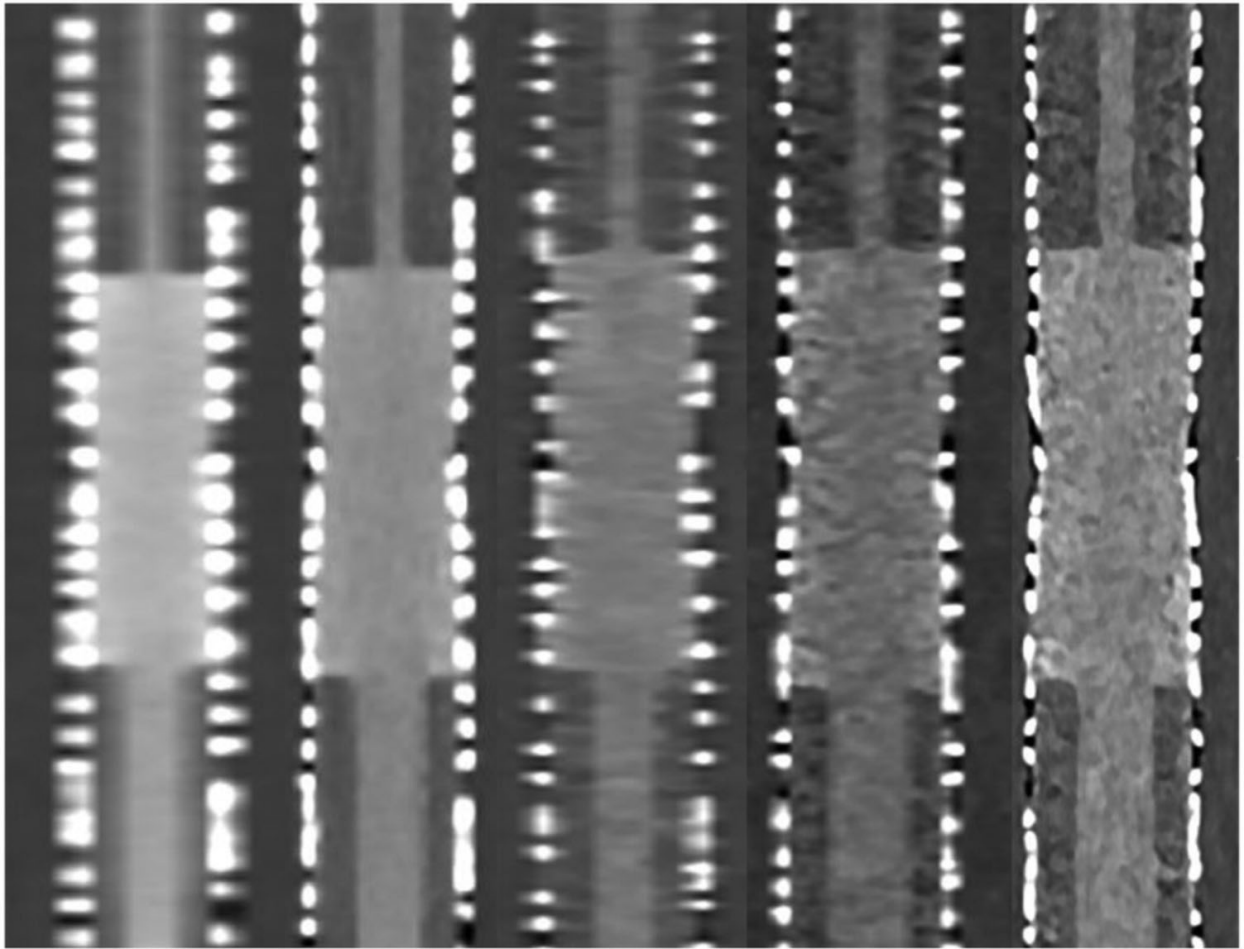
Visual impression of in-stent restenosis Comparison of an in-stent restenosis phantom with a stent caliber of 8 mm between (from left to right) energy-integrating detector CT at 1.0 and 0.5 mm slice thickness vs. photon-counting detector CT at 1.0, 0.5, and 0.2 mm slice thickness

Interrater agreement for qualitative analysis was good with an ICC of 0.75 [95% CI: [0.53; 0.86]. Mean sum scores calculated from the subjective assessments of two raters ranged from 17.79 to 19.43 of 20 possible. Both raters judged images of PCD-CT at 0.2 mm slice thickness best. Results are displayed in Table 3. A visual impression of images derived from the reported combinations of scanner type and slice thickness is provided in Fig. 3.

**Table 3:** Sum scores of qualitative analysis

Scanner	Slice thickness	Sum Score	
		Rater 1	Rater 2
EID	0.5	18.79 ± 0.43	18.64 ± 0.63
	1.0	18.36 ± 0.63	17.79 ± 1.05
PCD	0.2	19.43 ± 0.51	19.00 ± 0.68
	0.5	18.86 ± 0 .36	18.57 ± 0.51
	1.0	18.29 ± 0.99	18.29 ± 0.99

Number of observations = 14 in each setting

Two raters assessed images of energy-integrating detector computed tomography (EID-CT) and photon-counting detector computed tomography (PCD-CT) at varying slice thicknesses regarding 4 aspects (i.e., sharpness, subjective image noise, blooming, and diagnostic confidence). For each rater, sum scores were calculated. Possible values range from 0 to 20 points, whereas higher values indicate superior subjective image quality

## Discussion

In the present study, we compare clinically applied CTA protocols of UHR PCD-CT and third-generation dual-source EID-CT regarding the visualization of peripheral artery high- and low-grade ISRS in a phantom. We demonstrate high image quality of both modalities at a stent lumen of 8 mm, enabling a precise and therefore reliable, noninvasive differentiation between low- and high-grade ISRS.

In femoropopliteal disease, approximately one-third of patients are treated with stent implantation [23]. While postinterventional antiplatelet regimens decreased rates of stent thrombosis, intimal hyperplasia remains a significant complication of the procedure with up to 40% of ISRS within a two-year period [24, 25].

Current guidelines recommend duplex ultrasound (DUS) upon clinical suspicion of ISRS and digital subtraction angiography (DSA) where deemed necessary [10, 11, 26]. Besides, recent data suggest the additional usefulness of intravascular ultrasound and optical coherence tomography regarding ISRS lesion characterization (e.g., stent underexpansion, severely calcified lesions) [27, 28].

However, considering the general methodologic limitations of DUS (i.e., difficulties in anatomical access, reproducibility, documentation, operator’s experience), DSA (i.e., invasiveness, availability, cost), and intravascular imaging such as ultrasound and optical coherence tomography (i.e., invasiveness, availability, operator’s experience), CTA has been used as a diagnostic alternative in clinical practice with a growing body of supportive data [29–31]. Yun et al. examined 51 patients with a total of 68 stents and compared DSA to CTA with a dual-source EID-CT and reported an accurate detection of ISRS in EID-CT of 79% compared to DSA [8]. Furthermore, the first clinically approved PCD-CT has enabled improved image quality of coronary and peripheral stents in vitro and in vivo [5–7, 14, 17]. Overall, this development emphasizes the importance of further studies to test the accuracy of state-of-the-art CTA scanners in the depiction of previously stented vessels and the identification of ISRS.

In our study, we investigated seven widely used peripheral stents. Across all stent models, observed mean CNR was best in the 0% stenosis and lowest in the 95% stenosis, whereas findings of similar and even higher CNR in UHR PCD-CT compared to EID-CT may result from the improved contrast and lower image noise owing to the possibility of weighing photons differently, depending on their inherent energy [32].

Spatial resolution as measured by FWHM was high for both PCD-CT and EID-CT at any slice thickness, with deviations of the mean no larger than 0.5 mm from true lumen size. Combining the results for non-stenosed, significantly (i.e., 75%), and highly stenosed (i.e., 95%) segments, UHR PCD-CT at a slice thickness of 0.2 mm enabled estimations of lumen width with a precision (= half-width of CI) of 0.34 mm, 0.32 mm, and 0.15 mm, respectively.

We observed underestimation of the true lumen size in PCD-CT at slice thicknesses of 0.5 mm and 1.0 mm such as EID-CT at 1.0 mm. In highly stenosed segments, measurements of residual ISRS lumen diameter were prone to underestimation in both modalities, probably owing to lower CNR.

Qualitative assessment of images regarding sharpness, subjective image noise, blooming, and diagnostic confidence by two raters with basic and advanced experience levels displays good correlation and suggests high diagnostic usability (sum score from 17.79 to 19.43 of 20 possible) of both third-generation dual-source EID-CT and PCD-CT regarding ISRS in 8 mm caliber peripheral stents, whereas images of UHR PCD-CT at a slice thickness of 0.2 mm were rated best. Likely, it is the sum of improvements—lower image noise and higher CNR, enabled by weighing the energy of individual X-ray photons, such as better spatial resolution, achieved by PCD not necessitating reflective septa and respective smaller pixel sizes, that yield an overall improved visual impression of UHR PCD-CT images. The fact that orientation of the stents relative to the z-axis showed no detectable differences is attributable to the isotropic voxel sizes [7, 8].

We conclude that for the investigated stent lumen of 8 mm, both PCD-CT and state-of-the-art EID-CT yield good image quality to detect and accurately quantify ISRS, whereas the smallest slice thicknesses available for both scanners (i.e., 0.2 mm in PCD-CT vs. 0.5 mm in EID-CT) appeared advantageous, especially in high-grade ISRS. Our results stand in line with previous in vitro and in vivo data supportive of the reliably of noninvasive ISRS diagnosis in 8 mm and smaller diameter stents using both scanner types, in PCD-CT more so than EID-CT. The accuracy of both a third-generation dual-source EID-CT and PCD-CT is of clinical importance, especially given the currently limited availability of PCD-CT due to higher associated procurement costs. On another note, in the present study, PCD-CT and EID-CT acquisition parameters were matched using CTDI_vol_, underlining that the reported improvements in image quality do not come at the cost of excessive radiation. Finally, with prospects of software advancements such as experience in clinical settings, the potential of PCD-CT will certainly expand greatly in the foreseeable future.

The limitations of this study include, first, the in vitro nature, and thus, the absence of potential motion and pulsation artifacts, as well as beam hardening artifacts from bone or calcified tissue. In addition, the ideal contrast concentration inside the stents with the absence of potentially suboptimal filling of the lumen might have created a picture that is only obtainable under optimal clinical conditions and not in every real-world cases. Moreover, due to the comparison of clinically applied PCD-CT and EID-CT protocols with preselect kernels, we cannot exclude the possibility that other kernels might yield equal or even superior images of the presented ISRS-phantom. Last but not least, the study tested two CT scanners from a single manufacturer, which might limit the generalizability of results.

## Conclusion

In summary, we provide a detailed comparison of CTA protocols performed on the first clinically available UHR PCD-CT versus a third-generation dual source EID-CT in phantoms of peripheral artery ISRS. In stent diameters of 8 mm, our results argue for the good to excellent diagnostic usability of both scanners at the smallest slice thicknesses available. The observed tendency of vaguely superior spatial resolution and CNR in PCD-CT stands in line with previous observations in smaller caliber stents [14–16], however, seems negligible in case of larger stent sizes.

## Supplementary Information

Below is the link to the electronic supplementary material.

Supplementary file1 (DOCX 114 KB)
