# Supplementary material for: In-Stent Restenosis in Peripheral Arterial Disease: Ultra-High-Resolution Photon-Counting Versus Third-Generation Dual-Source Energy-Integrating Detector CT Phantom Study in Seven Different Stent Types
Source: Cardiovasc Intervent Radiol. 2024 Nov 5;48(1):65–74. doi: 10.1007/s00270-024-03874-y (PMC11706893; doi:10.1007/s00270-024-03874-y)
Supplement: Supplementary file 1 — Supplementary file1 (DOCX 114 KB) [file 270_2024_3874_MOESM1_ESM.docx]

SUPPLEMENTAL MATERIAL

**Table of contents**

[Table I. Overview of the stents used. 3](#_Toc165408457)

[Table II. Contrast-to-noise ratio of stent models. 3](#_Toc165408458)

[Figure I. Bland-Altmann plots of first and second measurements of full width at half-maximum. 4](#_Toc165408459)

# Table I. Overview of the stents used.

| Stent | Material | Strut Thickness, µm | Size*, mm |
| --- | --- | --- | --- |
| Absolute Pro (Abbott Vascular) | Nitinol | ~168 | 8x40 |
| Everflex (Medtronic) | Nitinol | ~215 | 8x60 |
| Epic (Boston Scientific) | Nitinol | ~181 | 8x60 |
| SMART Control (Cordis) | Nitinol | ~172 | 8x60 |
| Express LD (Boston Scientific) | Stainless steel | 178 | 8x57 |
| OmniLink Elite (Abbott Vascular) | CoCr | 141 | 8x59 |
| Dynetic-35 (Biotronic) | CoCr | 140 | 8x58 |

CoCr=Cobalt chromium alloy.

*Stent size is provided as diameter x length.

# Table II. Contrast-to-noise-ratio of stent models.

| Stent | Mean | SD | Med. | Q1 | Q3 | Min. | Max. |
| --- | --- | --- | --- | --- | --- | --- | --- |
| Nitinol | | | | | | | |
| Absolute Pro  (Abbott Vascular) | 8.50 | 2.43 | 8.79 | 6.39 | 9.83 | 4.84 | 13.43 |
| Everflex (Medtronic) | 8.41 | 2.61 | 7.92 | 6.31 | 10.13 | 4.77 | 13.00 |
| Epic (Boston Scientific) | 8.05 | 2.81 | 7.76 | 5.65 | 9.75 | 4.58 | 14.82 |
| SMART Control (Cordis) | 7.93 | 2.63 | 7.35 | 5.97 | 9.66 | 3.92 | 12.47 |
| Stainless steel | | | | | | | |
| Express LD (Boston Scientific) | 7.92 | 2.54 | 7.64 | 5.91 | 8.95 | 4.95 | 14.32 |
| Cobalt-chromium alloy | | | | | | | |
| OmniLink Elite (Abbott Vascular) | 7.81 | 2.58 | 7.33 | 5.86 | 9.31 | 4.77 | 13.44 |
| Dynetic-35 (Biotronic) | 6.66 | 2.34 | 6.37 | 4.97 | 7.93 | 3.74 | 11.95 |

Max.=maximum; Med.=median; Min.=minimum; Q1=first quartile; Q3=third quartile; SD=standard deviation.

# Figure I. Bland-Altmann plots of first and second measurements of full-width-at-half-maximum.


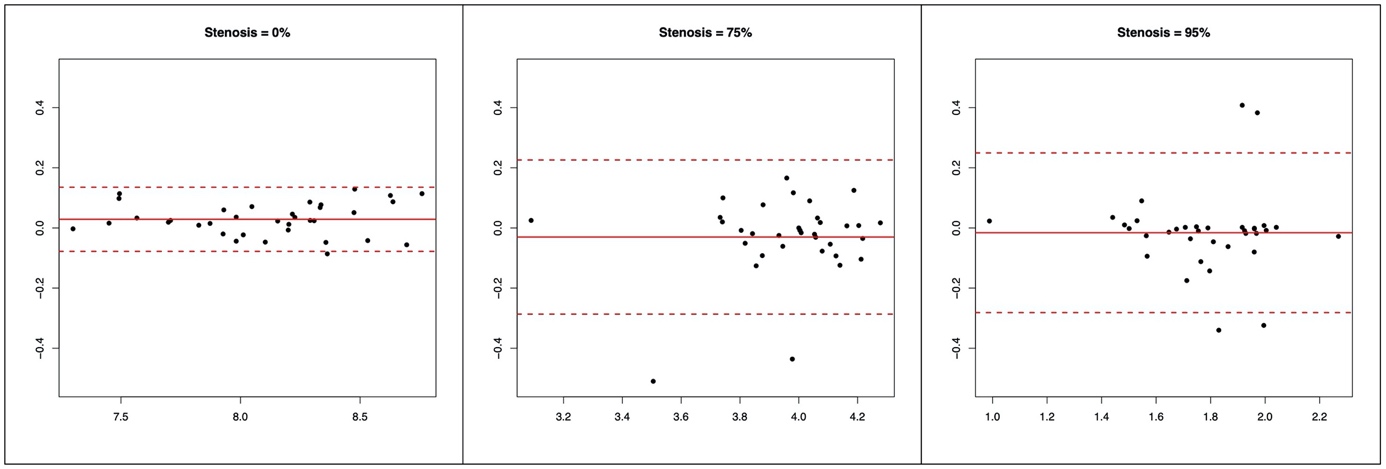


The difference between the two consecutive measurements of full-width-at-half-maximum is shown on the y-axis. The average of both measurements is shown on the x-axis. The solid red line shows the mean difference. The dashed red lines show the mean difference plus/minus the standard deviation of the differences x 1.96 (95% limits of agreement).
